# Stochastic sequence-level model of coupled transcription and translation in prokaryotes

**DOI:** 10.1186/1471-2105-12-121

**Published:** 2011-04-26

**Authors:** Jarno Mäkelä, Jason Lloyd-Price, Olli Yli-Harja, Andre S Ribeiro

**Affiliations:** 1 Computational Systems Biology Research Group, Department of Signal Processing, Tampere University of Technology, FI-33101 Tampere, Finland; 2 Institute for Systems Biology, 1441N 34th St, Seattle, WA, 98103-8904, USA

## Abstract

**Background:**

In prokaryotes, transcription and translation are dynamically coupled, as the latter starts before the former is complete. Also, from one transcript, several translation events occur in parallel. To study how events in transcription elongation affect translation elongation and fluctuations in protein levels, we propose a delayed stochastic model of prokaryotic transcription and translation at the nucleotide and codon level that includes the promoter open complex formation and alternative pathways to elongation, namely pausing, arrests, editing, pyrophosphorolysis, RNA polymerase traffic, and premature termination. Stepwise translation can start after the ribosome binding site is formed and accounts for variable codon translation rates, ribosome traffic, back-translocation, drop-off, and trans-translation.

**Results:**

First, we show that the model accurately matches measurements of sequence-dependent translation elongation dynamics. Next, we characterize the degree of coupling between fluctuations in RNA and protein levels, and its dependence on the rates of transcription and translation initiation. Finally, modeling sequence-specific transcriptional pauses, we find that these affect protein noise levels.

**Conclusions:**

For parameter values within realistic intervals, transcription and translation are found to be tightly coupled in *Escherichia coli*, as the noise in protein levels is mostly determined by the underlying noise in RNA levels. Sequence-dependent events in transcription elongation, e.g. pauses, are found to cause tangible effects in the degree of fluctuations in protein levels.

## Background

In prokaryotes, both transcription and translation are stochastic, multi-stepped processes that involve many components and chemical interactions. Several events in transcription and in translation [1-8] are probabilistic in nature, and their kinetics are sequence dependent. One example is sequence-dependent transcriptional pausing [1]. When they occur, these events can affect the degree of fluctuations of RNA and protein levels. Since noise in gene expression affects cellular phenotype, sequence dependent noise sources are subject to selection [9,10] and are thus evolvable [7]. Recent evidence suggests that these noise sources may be key for bacterial adaptability in unpredictable or fluctuating environmental conditions [11,12].

To better understand the evolvability of bacteria, it is important to understand how fluctuations in RNA levels propagate to protein levels. Transcription and translation are coupled in prokaryotes, in that translation can initiate after the formation of the ribosome binding site region of the RNA, which occurs during the initial stages of transcription elongation. The extent to which sequence-dependent events in transcription elongation affect the noise in RNA, and consequently protein levels is largely unknown. Due to this, it is also not yet well understood how phenotypic diversity is regulated in monoclonal bacterial populations.

Two recent experiments have given a preliminary glimpse at the dynamics of production of individual proteins [13] and RNA molecules [14]* in vivo *in bacteria. However, as of yet, there is no experimental setting to simultaneously observe the production of both RNA and proteins at the molecular level. Further, in the aforementioned experiments [13,14], the rate of gene expression was kept very weak, as otherwise the number of molecules would not be easily quantifiable. This implies that they cannot be used to study the effects of events such as the promoter open complex formation [15]. The present shortcomings of these techniques enhance the need for realistic models of gene expression in prokaryotes.

Several measurements have shed light on the dynamics of transcription and translation elongation [16,17], and revealed the occurrence of several stochastic events during these processes, such as transcriptional pauses [2,4]. The kinetics of RNA and protein degradation are also better known [18]. These measurements allowed the recent development of realistic kinetic models of transcription at the nucleotide level [5,19] and translation at the codon level [20]. These models were shown to match the measurements of RNA production at the molecule level [6,21] and of translation elongation dynamics at the codon level [20]. In this regard, it was shown that measurements of sequence dependent translation rates of synonymous codons could be modeled with neither deterministic nor uniform stochastic models [20], thus the need for models with explicit translation elongation. Similarly, transcription elongation also needs to be modeled explicitly to accurately capture the fluctuations in RNA levels for fast transcription initiation rates [5,19,22].

Here, we propose a model of transcription and translation at the nucleotide and codon level for *Escherichia coli*. The model of transcription is the same as in [5], and includes the promoter occupancy time, transcriptional pausing, arrests, editing, premature termination, pyrophosphorolysis, and accounts for the RNAp footprint in the DNA template. The model of translation at the codon level proposed here is based on the codon-dependent translation model proposed in [20], which includes translation initiation, codon-specific translation rates and the stepwise translation elongation and activation. The model also accounts for the ribosome's footprint in the RNA template as well as the occupancy time of the ribosome binding site. Here, beside these features, we further include the processes of back-translocation, drop-off, and trans-translation. Finally, we include protein folding and activation, as well as degradation, modeled as first-order processes, so as to study fluctuations in the protein levels.

The dynamics of the model follow the Delayed Stochastic Simulation Algorithm [19,23] and is simulated by a modified version of SGNSim [24]. While the most relevant innovation is the coupling between realistic stochastic models of transcription and translation at the nucleotide and codon levels, which allows the study of previously unaddressed aspects of the dynamics of gene expression in prokaryotes, this introduces a level of complexity that required simulation capabilities that SGNSim did not possess. Namely, the simulator is required to create and destroy compartments at run time within the reaction vessel, where a separate set of reactions can occur.

We start by validating the dynamics of translation elongation in the model. Next, using realistic parameter values extracted from measurements, we address the following questions: how different are the distributions of time intervals between translation initiation events and between translation completion events, i.e., how stochastic is translation elongation? To what extent do fluctuations in temporal RNA levels propagate to temporal protein levels, and what physical parameters control this propagation of noise between the two? Finally, we investigate whether transcriptional pauses have a significant effect on the dynamics of protein levels.

## Results and discussion

### Dynamics of transcript production

Given the number of chemical reactions per nucleotide in the model and that one gene can have thousands of nucleotides, the dynamics are considerably complex. To illustrate this, we show examples of the kinetics of multiple RNAps on a DNA strand within a short time interval, and the dynamics of multiple ribosomes on one of the RNA strands as it is transcribed. Parameter values were obtained from measurements in *E. coli *for *LacZ *(see methods section), since the dynamics of transcription and translation have been extensively studied for this gene. *LacZ *has 3072 nucleotides and its transcription is controlled by the lac operon.

In this simulation, transcription is not repressed. Thus, provided that the promoter is available for transcription, the expected time for a transcription event to start is approximately 2.5 s, given the value of the rate constant of reaction (1) in Table 1 and that there are 28 RNAp molecules available in the system [15]. The promoter open complex formation step, with a mean duration of 40 s [25] and a standard deviation of 4 s [21] is the major limiting factor of transcription events in these conditions.

**Table 1:** Reactions modeling transcription

Event	Reaction	Rate constant	**Ref**.
Initiation and promoter complex formation (1)		*k_init _*= 0.015 *τ_oc _*= 40 ± 4	[21]

Promoter clearance (2)		*k_m _*= 114	[37]

Elongation (3)		*k_m _*= 114	[37]

Activation (4)		*k_a _*= 114, n>10, *k_a _*= 30, n≤10	[37]

Pausing (5)		*k_p _*= 0.55 *τ_p _*= 3	[2]

Pause release due to collision (6)		*k_m _*= 114	[38]

Pause induced by collision (7)		*k_m _*= 114	[38]

Arrests (8)		*k*_*ar *_= 0.00028 *τ*_*ar *_= 100	[5]

Editing (9)		*k_ec _*= 0.008 *τ_c _*= 5	[2]

Premature termination (10)		*k_pre _*= 0.00019	[39]

Pyrophosphorolysis (11)		*k_pyro _*= 0.75	[40]

Completion (12)		*k*_*f *_= 2	[41]

mRNA degradation (13)		*k*_*dr *_= 0.011	[13]

Chemical reactions, rate constants (in *s^-1^*), and time delays (in *s*) used to model transcription initiation, elongation, and termination. Parameter values were obtained from measurements in *E. coli*, mainly for *LacZ*. References are reported in the column Ref.

**Figure 1:**
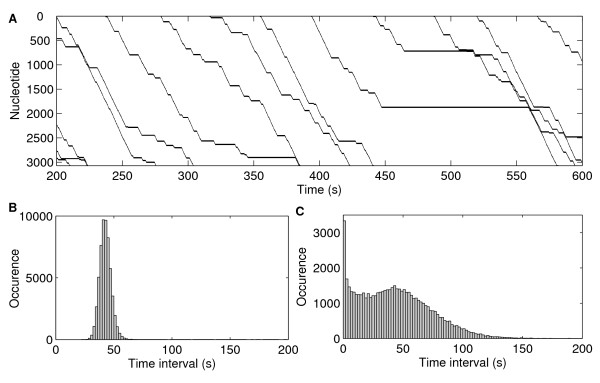
**Kinetics of RNA polymerases on the DNA strand**. (A) Example of the kinetics of multiple RNAp molecules on the DNA template over 400 s. Note that, on several occasions, the RNAp molecules pause and that one RNAp never overtakes another on the DNA template. (B) Distribution of time intervals between consecutive transcription initiation and (C) completion events. Data is from 57 000 initiation events.

Figure 1A shows, for a time window of 400 seconds, the positions (y-axis) over time (x-axis) of several RNAp molecules on the DNA template. In real time, this simulation takes ~30 s, on an Intel Core 2 Duo processor. Transcription elongation is visibly stochastic, with events such as arrests (e.g. at t = ~450 s), ubiquitous pauses and pyrophosphorolysis. Several collisions between RNAp molecules are also visible, caused in part by these events. Note that one RNAp never overtakes another on the template.

Figure 1B shows the distribution of the time intervals between transcription initiation events, which is Gaussian-like, due to the open complex formation step. The longer tail on the right side of the distribution is mainly due to the contribution of the time it takes for the RNAp to bind to the template, a bimolecular reaction whose expected time to occur follows an exponential distribution with a mean of 2.5 s [26,27].

Figure 1C shows the distribution of time intervals between transcription completion events in the same simulation as Figure 1B. This distribution is strikingly different from that of Figure 1B due to the stochastic events in transcription elongation. Pauses, arrests and other stochastic events cause the distribution to be bimodal due to the bursty dynamics (many short intervals and some long intervals). When these probabilistic events occur to some RNAp molecules, they significantly alter the distances in the strand between consecutive RNAps. For example, when one RNAp pauses, its distance to the preceding RNAp increases, while the distance to subsequent RNAps shortens, allowing completion events to be separated by intervals shorter than the promoter delay.

### Dynamics of production of proteins

**Figure 2:**
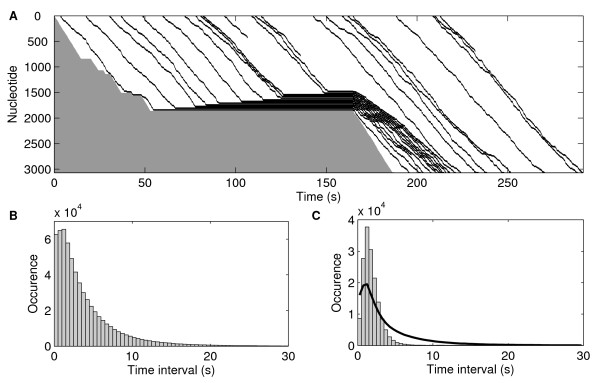
**Kinetics of ribosomes on an RNA strand**. (A) Example of the kinetics of several ribosomes along an mRNA template that suffered an arrest at nucleotide 1850, from the moment the ribosome binding site is formed to the degradation of the mRNA. The continuous gray region in the bottom left corresponds to the untranscribed sequence of the mRNA. (B) Distribution of time intervals between consecutive translation initiation events. (C) Distribution (grey bars) of time intervals between consecutive translation completion events given the presence of a sequence dependent arrest site at nucleotide 1850. The solid black line shows the distribution of time intervals between consecutive translation completion events without the sequence-dependent arrest site, normalized to the same scale. Data is from 600 000 initiation events.

Figure 2A exemplifies the dynamics of ribosomes on one RNA strand. Stochastically, the transcription elongation process of this particular mRNA was halted at t = 50 s for a long period, and was thus selected to illustrate how long pauses in transcription affect the dynamics of translation of the multiple ribosomes on the RNA strand. The solid gray region in the bottom left part of the figure corresponds to the as-of-yet untranscribed sequence of the mRNA. When the RNAp pauses or is arrested (e.g. at t = 50 s), ribosomes accumulate in the region of the mRNA preceding the leading edge of transcription. Stochasticity in the translation elongation process is also visible. However, this process, modeled with realistic parameter values, appears to be less stochastic than transcription elongation, in that the stepwise elongation of ribosomes on the RNA template is more uniform than that of the RNAps on the DNA template. This is especially visible after the effects of the long arrest disappeared (at t > 230 s), at which point the distributions of time intervals between consecutive ribosomes at the start and at the end of translation elongation do not differ significantly.

Figure 2B shows the distribution of intervals between translation initiation events. Since there is no significant delay in translation initiation (as the one due to the promoter open complex formation), this distribution is exponential-like. Figure 2C shows the corresponding distribution of intervals between translation completion events (grey bars), given the presence of a sequence dependent arrest site at nucleotide 1850. This distribution, while resembling that of Figure 2B, shows more short time intervals, due to the long arrest in transcription elongation. For comparison, we also show a distribution of intervals between translation completion events drawn from cases without the sequence dependent arrest in transcription (solid black line). The difference between the two distributions illustrates how events in transcription elongation (e.g. a sequence dependent arrest site) can significantly affect the dynamics of translation.

### Comparing the dynamics of the model of translation with measurements

Recently, the real-time expression of a lac promoter was directly monitored in *E. coli *with single-protein resolution [13]. The proteins were found to be produced in bursts (i.e. several proteins being produced from each RNA), with the distribution of intervals between bursts fitting an exponential distribution, while the number of proteins per burst followed a geometric distribution [13]. These distributions were measured for a gene that was kept strongly repressed and for which the ribosome binding site (RBS) was engineered so that translation was also very weak [13]. Under these conditions, our model reproduces these dynamics (data not shown). Nevertheless, we note that it is possible to match these measurements with a simpler model than the one proposed here, where transcription and translation are modeled as single step events [21,23].

We next compare the kinetics of translation in our model with measurements of the translation elongation speed in three engineered *E. coli *strains designed to enhance queue formation and traffic in translation [17]. Each strain contains a different mutant of *LacZ*. The pMAS23 strain corresponds to the wild-type *lacZ*. The other two sequences differ in that a region of slow-to-translate codons was inserted (~24 in pMAS-24GAG and ~48 in pMAS-48GAG). The speed of protein chain elongation was measured by subjecting the cells to a pulse of radioactive methionines, and then measuring the level of radioactivity in cells of each population, every 10 s after the pulse. Each strand contained 23 methionines, spread out unevenly on the DNA sequence, causing the incorporation curve to be non-linear.

Given that they differ in the nucleotide sequence, it was hypothesized that the translation elongation speed of the three strands would differ, as the speed of incorporation of an amino acid depends on which synonymous codon is coding for it [17]. The cells where translation is faster will thus be expected to have higher levels of radioactivity in the translated proteins, as more labeled amino acids have been incorporated in a fixed time interval. If the translation speeds of the three strands were identical, they would exhibit identical levels of radioactivity at the same point in time.

**Figure 3:**
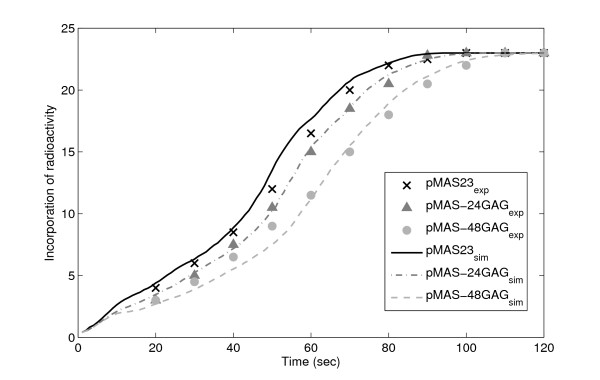
**Appearance of radioactivity in β-galactosidase**. Appearance of radioactivity incorporated from the three different mRNA strands, at different times after initiation of translation elongation in the models (lines) and in the measurements (crosses, triangles and circles) [17]. Values of radioactivity are normalized such that the maximum corresponds to 23 radioactive methionines.

To model this, we simulate the transcription and translation processes of the three sequences [17]. We model the incorporation of radioactive methionines at the same locations as in these sequences. The three model strands differ only in sequence, as in the measurements. During the simulations, we measure the number of incorporated radioactive methionines at the same points in time as in the experiment. Results of our simulations and of the measurements [17] are shown in Figure 3, showing good agreement between model and measurements.

### Propagation of fluctuations in RNA levels to protein levels

We simulate the model for varying effective rates of transcription initiation (denoted k_eff_). This rate is determined by the basal rate of transcription initiation (k_init_), which sets the binding affinity of the RNAp to the transcription start site, and by the strength of repression of transcription. Thus, to vary k_eff_, we vary the number of repressor molecules present in the system. Three sets of simulations are performed, differing in rate of translation initiation (k_tr_). This rate is one of the kinetic parameters of the model, thus can be changed directly, and not by indirect means as k_eff_. In *E. coli *genes, this rate is believed to be determined by the RBS sequence [28]. mRNA and protein degradation rates are set so that the mRNA and protein mean levels are identical for all cases, allowing us to study how the level of noise in mRNA and protein levels changes.

For each set of values of k_eff _and k_tr _we perform 100 independent simulations. Depending on these rates, the mean time to reach steady state differs. Each case is simulated for long enough to reach steady state and for an additional 100 000 s after that. The time series of the 100 simulations for each set of parameter values is concatenated into one time series, from which the noise is quantified by the square of the coefficient of variation, CV^2 ^(variance over the mean squared) [29]. This number of long simulations is necessary to properly sample the system due to the stochasticity of the underlying processes.

**Figure 4:**
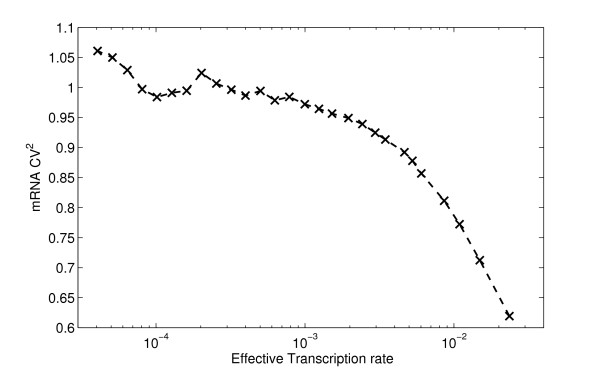
**Noise in mRNA as a function of the transcription initiation rate**. Noise (CV^2^) in mRNA levels for varying effective transcription initiation rates. The mRNA degradation rate is set so that the mean mRNA levels at steady state are identical in all cases.

In Figure 4, we first show the CV^2 ^of mRNA time series for varying k_eff_. Noise decreases as k_eff _increases due to the promoter open complex formation step [6]. Without this event, the distribution of time intervals between transcription initiation events would be exponential, and the CV^2 ^would not vary. However, with this step, if the expected time for an RNAp to bind to the free promoter is faster than the duration of the promoter open complex formation, then the distribution of time intervals becomes Gaussian-like [6].

**Figure 5:**
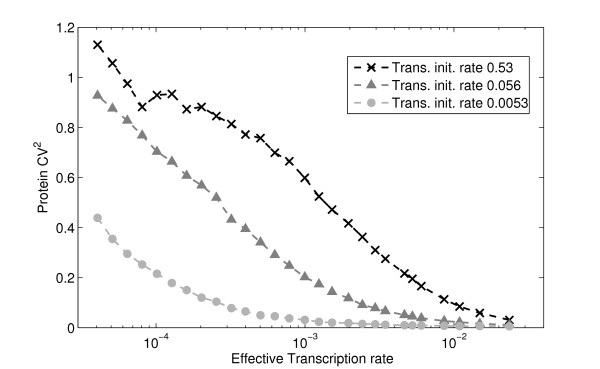
**Noise in protein levels for varying transcription and translation initiation rates**. Noise (CV^2^) in protein levels for varying effective transcription initiation rates and three different rates of translation initiation. mRNA and protein degradation rates are set so that the mean mRNA and mean protein levels at steady state are identical in all cases.

No measurements have yet been made to study experimentally the relation between the noise in mRNA levels and the corresponding protein levels. Nevertheless, it is possible to create a robust estimate, provided reasonable assumptions on the nature of the underlying processes [8]. Our model allows for a direct assessment, and it additionally includes realistic events such as RNAp and ribosome traffic, in transcription and translation elongation, which are not included in the aforementioned estimations [8]. Figure 5 shows the noise (CV^2^) in protein levels, for varying k_eff _and three values of k_tr_. The data was obtained from the same simulations used to generate the results in Figure 4.

In general, we find that increasing k_eff _decreases the noise in protein levels due to the decrease of noise in mRNA levels. Increasing k_tr _increases the noise in protein levels, due to the increased size of the bursts in the protein level [8,29]. This finding has not yet been experimentally validated by direct means.

An interesting observation from Figures 4 and 5 is that, for k_eff _< 5 × 10^-4 ^s^-1^, as k_eff _is increased, the noise in protein levels decreases significantly, while the noise in RNA levels does not noticeably change. This is due to the decrease in mean protein burst size, i.e., the mean number of proteins produced from each RNA molecule, as both k_eff _and the degradation rate of RNA molecules are varied.

**Figure 6:**
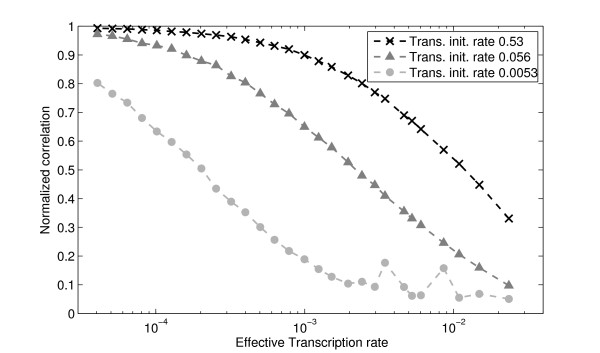
**Normalized maximum correlation between RNA and protein time series**. The higher the rate of translation initiation (and thus higher protein degradation to keep the mean the same), the more correlated the fluctuations in protein and RNA levels become, as measured by the normalized maximum correlation. This is because the protein levels follow any fluctuations in the RNA levels faster. Similarly, increasing the rate of transcription initiation, while maintaining the rate of translation initiation constant, decreases the correlation between fluctuations in protein and RNA levels.

From these results, we conclude that the degree of coupling between transcription and translation is likely to be a key determining factor of the noise in protein levels. This can be verified by computing the normalized maximum correlation between time-series of protein and mRNA levels for each set of parameter values (Figure 6). Comparing Figures 5 and 6, we see that higher correlation values are obtained for the regime of higher noise in the protein levels. This implies that the principal source of this noise is the fluctuations in RNA levels.

The correlation value is largely determined by the rates of mRNA and protein degradation and production. For example, both increasing the mRNA degradation rate and/or decreasing the protein degradation rate increases the time averaging constant of the mRNA fluctuations, and thus decreases the correlation between mRNA and protein levels. In general, if the mean mRNA and protein levels and kept unchanged by tuning their degradation rates accordingly, the correlation between RNA and protein time series can be increased by lowering the mRNA production rate and/or increasing the protein production rate.

### Effects of transcriptional pauses on the fluctuations in protein levels

Recent work [1] reported that long transcriptional pauses enhance the noise in mRNA levels. We next investigate to what extent the fluctuations in RNA levels caused by long transcriptional pauses propagate to protein levels. Long sequence-dependent pauses [16,30,31] in transcription elongation may cause the ribosome to stall in the mRNA chain. This will likely cause subsequent ribosomes to accumulate in the preceding sequence. When the RNAp is spontaneously released from the pause [31], translation of the stalled ribosomes likely resumes but the distribution of intervals between them will differ significantly from what it would have been without the pause event. Consequently, the protein production is likely to become burstier, especially if the long pause site is located near the end of the sequence. An increase in burstiness ought to increase the noise in protein levels.

To verify this, we perform two simulations. We introduce a long-pause sequence with mean pause durations of 500 s in one case, and 100 s in the other (both values are within realistic intervals [30]). In both cases, we set the probability that an RNAp will pause at that site to 70% (identical to the value for *his *pause sites [16]).

Measuring the protein noise levels, we find that the CV^2 ^is ~5% higher for the 100 s pause site and ~10% higher for the 500 s pause site, in comparison to the same sequence without any sequence specific long-pause site. These relative differences can be biologically relevant in that such a change may, in some cases, cause the degree of phenotypic diversity of a monoclonal cell population to change.

The effects of several pause sites on the same strain are cumulative, namely, the higher the number of pause sites, the higher the noise in RNA levels [32]. Combined with the present results, this leads us to the conclusion that the sequence-dependent transcriptional pausing mechanism likely exists to allow a wide variation of both RNA and protein noise levels.

## Conclusions

We proposed a new delayed stochastic model of prokaryotic transcription and translation at the single nucleotide and codon level, where the processes of transcription and translation are dynamically coupled in that translation can initiate immediately upon the formation of the ribosome binding site region of the nascent mRNA. Simulations of the model's dynamics show that, within realistic parameter values, the protein noise levels are determined, to a great extent, by the fluctuations in the RNA levels, rather than from sources in translation, in agreement with indirect measurements [14], as translation elongation was found to be less stochastic than transcription elongation. Specifically, the distributions of intervals between translation initiation and translation completion events only differ significantly if the sequence possesses long sequence-dependent pauses or clusters of slow-to-translate codons. The sequence dependence of several mechanisms that can act as generators of strong fluctuations in RNA levels [15], the propagation of these fluctuations to protein levels, and the ability of fluctuations in protein levels to affect cellular phenotype [33], suggest that these mechanisms may be evolvable.

As a previous study has suggested [8], the translation initiation rate was found to be key in determining the degree of coupling between the fluctuations in RNA and protein levels, if one assumes that the degradation rate of the proteins is changed accordingly to maintain their mean level unchanged. Varying this sequence-dependent, and thus, evolvable parameter [28] within realistic ranges gave a widely varying degree of coupling between the fluctuations in RNA and protein levels. It is therefore not necessarily true that noisy production of RNA molecules results in noisy protein levels. Interestingly, while decreasing the coupling between transcription and translation by decreasing the rate of translation initiation causes the protein levels to become less noisy, it also takes longer for a change in RNA levels to be followed by the protein levels. This suggests that to be able to change rapidly in response to, e.g., environmental changes, the levels of a protein will be necessarily noisier.

Confirming previous studies [1,5,8,19], we found that the distributions of time intervals between transcription initiation and completion events differ significantly and that the faster the rate of transcription initiation events, the more they differ. This implies that in the regime of fast transcription, both the transcription and translation elongation processes need to be modeled explicitly and coupled, if one is to match the mean and fluctuations in the protein levels at the molecular level. This is of relevance, since bursts in protein levels may trigger many processes, such as phenotypic differentiation [33,34]. A final justification for using the model proposed here is the complexity of the process of gene expression in *E. coli*, and the fact that many events therein may or may not affect the temporal RNA and protein levels significantly, depending on their specific sequence-dependent features. Such effects, due to the complexity of the system, are not easily predictable without performing explicit numerical simulations.

The model proposed here includes several features not included in previous models such as a gradual degradation event that can be triggered while the RNA is still being transcribed. As its parameter values were extracted from measurements, it should be useful in the study of several aspects of the dynamics of gene expression in prokaryotes that cannot yet be measured directly and to explore the state space of gene expression dynamics by varying any of the physical variables within realistic ranges.

However, the present model does not yet account for known effects of ribosomes on the dynamics of transcription elongation. These might need to be included in future developments of the proposed model as recent results [27,35] suggest that the rate of translation elongation can affect the rate of transcription elongation, due to possible interactions between the ribosome that first binds to the mRNA and the RNAp transcribing it. Possible effects may include facilitating the release of paused RNAp's, which could affect the degree of the contribution of pauses to the noise in RNA and thus protein levels. We do not exclude the possibility that the contrary may occur in specific cases, that is, that the paused state of the RNAp may cause pauses in the ribosome translational dynamics, which would amplify the effect of transcriptional pauses on the fluctuations of protein levels. Whether the pause is ubiquitous or due to loop formations in the nascent RNA may affect the results of the interaction as well. Provided experimental evidence on the nature and consequences of these interactions, once included in the model, we may be able to test, among other things, whether long transcriptional pauses located in an attenuator system provide an additional layer of control over premature transcription terminations, and thus over RNA and protein noise levels.

## Methods

### Model of transcription, one nucleotide at a time

We model the dynamics of gene expression as in [23]. This model was shown [21] to match the dynamics of RNA and protein production at the single molecule level [13]. The dynamics of the system of chemical reactions is driven by the delayed stochastic simulation algorithm (delayed SSA [19]) so as to include events whose time of completion once initiated is non negligible, in that it affects the dynamics of production of RNA and protein molecules. Specifically, several steps in gene expression, such as the promoter open complex formation, are time consuming [36]. To include these events when simulating gene expression, the delayed SSA was proposed [19].

All simulations are executed by an extended version of SGNSim [24] to allow multiple coupled chain elongation processes to run in parallel on each elongating RNA strand. The extension consists in providing the simulator with the ability to introduce new chemical reactions at run time (that is, those corresponding to the translation of each individual RNA strand).

The delayed stochastic model of transcription at the nucleotide level [5] includes the promoter occupancy time, pausing, arrests, editing, premature terminations, pyrophosphorolysis, and accounts for the RNAp footprint in the DNA template [2]. Additional reactions model the stepwise forward movement and activation of the RNAp, pausing and unpausing of the RNAp due to collisions with adjacent RNAps, release of the promoter when the RNAp begins elongation, and error correction.

The reactions, stochastic rate constants and time delays, are shown in Table 1 and described in detail in [5,37-41]. Here, Pro stands for the promoter region, RNAp for the RNA polymerase, and RNAp·Pro for the promoter region occupied by an RNAp. A*_n_*, O*_n _*and U*_n _*stand for the *n*th nucleotide when activated, occupied, and unoccupied, respectively. Ranges of nucleotides are denoted such as U_[*start*, *end*]_, denoting a stretch of unoccupied nucleotides from indexes *start *to *end*. ,  and  are used to represent a paused, arrested, or error correcting RNAp at position *n*. On the template, each RNAp occupies (2Δ_RNAp_+1) nucleotides, where Δ_RNAp _= 12. These nucleotides cannot be occupied by any other RNAp at the same time.  denotes transcribed ribonucleotides which are free (i.e., not under the RNAp's footprint). These transcribed ribonucleotides are created in a separate part of the simulation (denoted by the R superscript), one separate set per RNA strand, so that we can simulate the translation of all individual RNA molecules independently and simultaneously.

We use a delayed reaction event to model the first step in transcription, the promoter closed and open complex formation (1). These processes could instead be modeled by a set of non-delayed, consecutive, reactions [42]. We use a delayed reaction as it was shown to accurately model the dynamics of this process [19,21,23]. The duration of this step likely varies from one event to the next, but while values for the mean duration are known, as of yet, there are no exact measurements of the standard deviation. Nevertheless, it is likely small compared to the mean, given the very small standard deviations of promoter activity [25]. For these reasons, we set the promoter delay, *τ*_*oc*_, as a random variable, following a normal distribution with a mean of 40 s and a standard deviation of 4 s, whose value is randomly drawn each time a transcription event occurs.

Once the first nucleotide is occupied via reaction (2), stepwise elongation can begin (3). Also, as soon as the promoter is released, a new transcription initiation event can occur. Following each elongation step (3), an activation step occurs (4), which is necessary for the RNAp to move along the template to the next nucleotide. The following events compete with stepwise elongation: pausing (5) and (7), released via (5) or (6), arrests and their release (8), editing (9), premature terminations (10), and pyrophosphorolysis (11).

At the end of the elongation process, the RNAp is released (12). mRNA degradation is modeled, for simplicity, as a first order reaction (13). When (13) occurs, the first few ribonucleotides of the RNA are immediately removed from the system, preventing any new translation event [43]. Thus, we model the degradation process such that it begins in the vicinity of the RBS and then gradually cuts the mRNA as it is being released from the ribosomes. This allows the translating ribosomes to complete protein production before the whole mRNA is degraded. When the final ribosome unbinds from the RNA, the rest of the RNA strand, denoted by R in reaction (13), is destroyed.

If the model of RNA degradation was such that some of the ribosomes on the RNA template fell off when degradation begins (i.e. due to endonucleatic cleavage of the RNA chain at a random position [43]), one consequence would be the reduction of the mean protein burst size as these RNAs would contribute far fewer proteins than if the ribosomes were allowed to finish translating. This would likely result in a reduction of protein noise levels. Alternatively, the ribosome occupancy of the ribosome binding site might determine mRNA longevity [28]. In this case, for the same mean burst size, the noise is expected to increase since large bursts will get larger and small bursts will get smaller, likely increasing protein noise levels. We opted not to include these additions to the degradation model since they are not yet well characterized [43].

Finally, we note that in present model we do not add an explicit reaction for abortive initiation of transcription [44]. This could be done by adding a reaction (2b) which would compete with reaction (2). Its rate, *k_ab_*, would be set so as to match the fraction of abortive initiations after the formation of the promoter open complex [44]:(2b)

For simplicity, we opted not to include this reaction in the simulations, and instead set a value for the rate of transcription initiation that matches realistic rates of RNA production. From the point of view of RNA production, since (2b) competes with reaction (2), it would be dynamically equivalent to decrease the rate of transcription initiation in (2) to account for the fraction of abortive initiations.

The model of transcription and the reaction rates in Table 1 are described in greater detail in [5]. Parameter values were obtained from measurements in *E. coli*, mainly for *LacZ*.

### Model of translation, one codon at a time

The stochastic model of translation at the codon level includes initiation (14) and stepwise translocation (codon incorporation) (15-17) followed by activation (18). Reactions competing with translocation are back-translocation (19), drop-off (20), and trans-translation (21). The process ends with elongation completion (22), followed by protein folding and activation (23). Protein degradation (24) is included to allow us to study fluctuations in protein levels at steady state. All reactions and rate constants are presented in Table 2[45-47]. Here, Rib denotes a free ribosome complex in the cellular medium, while Rib^R ^denotes a ribosome bound to a specific RNA strand. Similar to Δ_RNAp_, Δ_Rib _denotes the ribosome's footprint in the RNA template. Each ribosome occupies (2Δ_Rib_+1) ribonucleotides, where Δ_Rib _= 15 [20]. ,  and  are the ribonucleic equivalents of U*_n_*, O*_n _*and A*_n_*.  denotes an unoccupied ribonucleotide, while  denotes that a translating ribosome is currently positioned at ribonucleotide *n*. Similarly,  denotes that a ribosome has created peptide bond for the peptide coded by the codon at position [*n*-2,*n*], where *n *is a multiple of 3 (*n *= 3, 6, 9,...). Since different codons are translated at different rates, the activation reaction has a codon-specific rate [17]. Specific rates were set for four codons, while the remaining ones fall into three different classes [20], A, B and C, whose rates are denoted *k_trans{A, B, C}_*.

**Table 2:** Reactions modeling translation

Event	Reaction	Rate constant	**Ref**.
Initiation (14)		*k_trans_init _*= 0.33	[20]

Stepwise translocation (15-17)		*k_tm _*= 1000	[3]

Activation (18)		*k_transA_*= 35, *k_transB_*= 8, *k_transC_*= 4.5	[20]

Back-translocation (19)		*k_bt _*= 1.5	[51]

Drop-off (20)		*k_drop _*= 0.000114	[45]

Trans-translation (21)		*k_tt _*= 0.000052	[46]

Elongation completion (22)		*k_trans_f _*= 2	[20]

Folding and activation (23)		*k_fold _*= 0.0024	[47]

Protein degradation (24)		*k_dec _*= 0.0017	[47]

Chemical reactions and rate constants (in *s^-1^*) used to model translation initiation, elongation, and termination, as well as protein folding and activation, and protein degradation. Parameter values were obtained from measurements in *E. coli*, mainly for *LacZ*. References are reported in the column Ref.

Translation has three main phases: initiation, elongation and termination. It begins with the binding of the ribosome complex to the mRNA strand. During elongation, the amino acids, determined by the RNA sequence, are added to the elongating peptide chain. Termination is the final step, as specific release factors detach the peptide and the RNA chain from the ribosome. *E. coli *has specific translation factors for each phase: initiation factors IF1, IF2 and IF3, elongation factors EF-G, EF-Tu and EF-Ts and three release factors RF1, RF2 and RF3 [48]. These are not explicitly modeled, as they exist in abundance under normal conditions.

The binding of the ribosome to the ribosome binding site (RBS) of the RNA starts with the binding of the 30S ribosomal subunit to the nascent mRNA. After that, fMet-tRNA binds to the P-site forming a 30S complex. The 50S ribosome subunit attaches to it, forming the 70S initiation complex [48]. This process is modeled as a single step reaction (14). The next ribosome can only to bind after the preceding one has moved away from the RBS. This implies that the initiation of two consecutive translation events is separated by a non-negligible time interval.

Translation elongation occurs through successive translocation-and-pause cycles [3]. Translocation includes three steps (15-17), after which there is a pause (18), during which the bond between amino acids is formed. The time that (18) takes to occur accounts for this pause, which is much longer than the time for (15-17) to occur [3].

The genetic code contains two mechanisms for redundancy: some tRNAs can be charged with the same amino acid, and a single tRNA can recognize more than one codon due to a "wobble" effect in position three of the anti-codon [48]. The net effect is that multiple codons code for the same amino acid. These codons are called synonymous codons. Synonymous codons read by the same tRNA have been shown to translate at significantly different rates [17], implying that our model must incorporate per-codon translation rates for reaction (18), rather than per-tRNA or per-amino acid rates. Only a few of these translation rates have been measured directly [17] but indirect assessment is available [20]. In our case, we assume normal cellular conditions, including an abundance of charged tRNA, implying that we do not need to model the tRNA explicitly.

Since each codon is translated at a different rate, the codon frequency also needs to be accounted for explicitly [49]. In the model, the sequence can either be randomly generated or selected from a known gene. In the former case, the sequence is randomly generated according to the known statistical frequency of each codon in *E. coli*.

The competing reactions of stepwise translation elongation are back-translocation (19), drop-off (20) and trans-translation (21), which are explicitly modeled. Back-translocation generally occurs when the tRNA has not yet locked into the peptide chain, causing the ribosome to move backwards on the mRNA template to the position of the previous codon. While the occurrence of back-translocation has been observed and can be promoted by certain antibiotics [50-52], its exact causes remain somewhat unknown. Nevertheless, the kinetic rates for translocation and back-translocation have been measured under various conditions [51]. Alternatively, the ribosomes can randomly dissociate from the RNA, in a process called drop-off, modeled by reaction (20). The overall rate of drop-off has been measured in [45], from which we have inferred a per-codon rate.

Trans-translation is the process by which the ribosome is released from the RNA template after stalling, which can occur for a variety of reasons, such as the incorporation of an incorrect codon, premature mRNA degradation, or spontaneous frameshifting [53]. Trans-translation is executed by the tmRNA that, together with SmpB and EF-Tu, binds to the A-site of the ribosome and releases it from the mRNA [53]. Once the ribosome is released, the mRNA is degraded. In the model, stalling followed by trans-translation can occur spontaneously with a given probability at any codon via reaction (21). When this reaction occurs, the RNA strand is immediately destroyed in the simulation, and all translating ribosomes are released back into the cellular medium, denoted in reaction (21) by [Rib^R^]Rib, where [Rib^R^] denotes the number of ribosomes bound to the RNA at that moment.

Translation elongation continues until the STOP codon is reached (22), after which RF1 or RF2 binds and releases the ribosome together with RF3 [48]. These are not modeled explicitly in the model. Its kinetic rate is higher than initiation, preventing queuing near the stop codon [20]. Reaction (22) is followed by folding and activation (23), modeled as a first order process for simplicity [21]. The rate of this reaction is set to model the maturation time of GFP, as most measurements of protein expression at the single cell level use this protein. P_prem _denotes the unfolded protein, while P denotes the complete activated protein, which can then degrade via reaction (24).

Given the above, we note that the dynamics of transcription and translation are sequence dependent in the present model in the following ways. First, the model allows the insertion of, e.g., arrests or sequence specific pauses at a specific nucleotide (exemplified in the last section of the results section). In general, since the rates of all possible events are defined uniquely for each nucleotide, any event may be set to have a distinct propensity at a specific nucleotide rather than a constant rate for all nucleotides. Translation elongation is, in the same manner, sequence dependent, with the additional feature that the rates of elongation in this case are always codon dependent.

The chemical reactions and rate constants (in *s^-1^*) used to model translation initiation, elongation, and termination, as well as protein folding and activation and protein degradation are in Table 2. Parameter values were obtained from measurements in *E. coli*, mainly for *LacZ*.

### Quantifying the correlation between protein and mRNA levels

Protein levels do not respond instantaneously to changes in the number of mRNA molecules in the system since new proteins take time to synthesize after a new mRNA is produced, and excess proteins take time to degrade after an mRNA has been degraded. Instead, the fluctuations in protein levels result from a time averaging of the fluctuations in mRNA levels [8]. The degree to which fluctuations propagate from RNA to protein levels depends on various parameters, the most relevant being the ratio between the degradation rates of the proteins and RNAs. Changing this ratio is likely to affect the degree of correlation between the RNA and protein time series.

To assess the extent to which fluctuations in RNA levels are propagated to protein levels, we compute the normalized discrete cross-correlation [54] between the time series of RNA and protein numbers. The normalized cross-correlation function *r *for *m *pairs of time series (*x *and *y*) of discrete signals of length *n *is given by:(25)

where τ ∈ {0,..., n-1} is the lag, and *m*_w _and *s*_w _are the sample mean and sample standard deviation of *w*, respectively, defined by:(26)(27)

## Authors' contributions

All authors contributed in the design of the study, data acquisition and interpretation, and participated in the drafting of the article. All authors have read and approved the final manuscript.
